# Seroprevalence of anti-SARS-CoV-2 IgG in asymptomatic and pauci-symptomatic people over a 5 month survey in Argentina

**DOI:** 10.26633/RPSP.2021.66

**Published:** 2021-06-21

**Authors:** Luz María Rodeles, Luz María Peverengo, Romina Benítez, Nadia Benzaquen, Priscila Serravalle, Ana Karina Long, Virginia Ferreira, Agostina Daiana Benitez, Luisina Zunino, Camila Lizarraga, Miguel Hernán Vicco

**Affiliations:** 1 Universidad Nacional del Litoral Santa Fe Argentina Universidad Nacional del Litoral, Santa Fe, Argentina.; 2 Centro de Especialidades Médicas Santa Fe Argentina Centro de Especialidades Médicas, Santa Fe, Argentina.; 3 Sanatorio Diagnóstico Santa Fe Argentina Sanatorio Diagnóstico, Santa Fe, Argentina.

**Keywords:** Seroprevalence, SARS-CoV-2, asymptomatic diseases, anosmia, epidemiology, Argentina, Estudios seroepidemiológicos, SRAG-CoV-2, enfermedades asintomáticas, anosmia, epidemiología, Argentina, Estudos soroepidemiológicos, SRAG-CoV-2, doenças assintomáticas, anosmia, epidemiologia, Argentina

## Abstract

**Objective.:**

To evaluate the seroprevalence of COVID-19 infection in pauci-symptomatic and asymptomatic people, the associated epidemiological factors, and IgG antibody kinetic over a 5-month period to get a better knowledge of the disease transmissibility and the rate of susceptible persons that might be infected.

**Methods.:**

Seroprevalence was evaluated by a cross-sectional study based on the general population of Santa Fe, Argentina (non-probabilistic sample) carried out between July and November 2020. A subgroup of 20 seropositive individuals was followed-up to analyze IgG persistence. For the IgG anti-SARS-CoV-2 antibodies detection, the COVID-AR IgG^®^ ELISA kit was used.

**Results.:**

3 000 individuals were included conforming asymptomatic and pauci-symptomatic groups (n=1 500 each). From the total sample, only 8.83% (n=265) presented reactivity for IgG anti-SARS-CoV-2. A significant association was observed between positive anti-SARS-CoV-2 IgG and a history of contact with a confirmed case; the transmission rate within households was approximately 30%. In the pauci-symptomatic group, among the seropositive ones, anosmia and fever presented an OR of 16.8 (95% CI 9.5-29.8) and 2.7 (95% CI 1.6-4.6), respectively (p <0.001). In asymptomatic patients, IgG levels were lower compared to pauci-symptomatic patients, tending to decline after 4 months since the symptoms onset.

**Conclusion.:**

We observed a low seroprevalence, suggestive of a large population susceptible to the infection. Anosmia and fever were independent significant predictors for seropositivity. Asymptomatic patients showed lower levels of antibodies during the 5-month follow-up. IgG antibodies tended to decrease over the end of this period regardless of symptoms.

## RESUMEN

**Objetivo.:**

Evaluar la seroprevalencia de la infección por el virus causante de la COVID-19 en personas paucisintomáticas y asintomáticas, los factores epidemiológicos asociados y la cinética de los anticuerpos IgG durante un período de cinco meses para conocer mejor la transmisibilidad de la enfermedad y la tasa de personas susceptibles a estar infectadas.

**Métodos.:**

Se evaluó la seroprevalencia mediante un estudio transversal basado en la población general de Santa Fe, Argentina (muestra no probabilística) llevado a cabo entre julio y noviembre del 2020. Se realizó un seguimiento de un subgrupo de 20 personas seropositivas para analizar la persistencia de los anticuerpos IgG. Para la detección de los anticuerpos IgG contra SARS-COV-2, se empleó el kit ELISA COVID-AR IgG^®^.

**Resultados.:**

Hubo 3 000 participantes divididos en un grupo asintomático y un grupo paucisintomático (n=1 500 cada grupo). De la muestra total, solo 8,83% (n=265) presentó una reactividad de IgG contra el SARS-CoV-2. Se observó una asociación significativa entre anticuerpos IgG positivos contra el SARS-CoV-2 y antecedente de contacto con un caso confirmado. La tasa de transmisión en el hogar fue de 30% aproximadamente. En el grupo paucisintomático, entre las personas seropositivas, la anosmia y la fiebre presentaron un OR de 16,8 (IC 95% 9,5-29,8) y 2,7 (IC 95% 1,6-4,6), respectivamente (p <0,001). En los pacientes asintomáticos, los niveles de IgG fueron inferiores en comparación con los pacientes paucisintomáticos, con tendencia a la baja pasados cuatro meses desde la aparición de los síntomas.

**Conclusiones.:**

Se observó una seroprevalencia baja, indicadora de una gran población susceptible a la infección. La anosmia y la fiebre fueron factores predictivos independientes de relevancia para la seropositividad. Los pacientes asintomáticos mostraron niveles inferiores de anticuerpos durante el seguimiento de cinco meses. Los anticuerpos IgG tendieron a disminuir hacia el final del período con independencia de los síntomas.

## RESUMO

**Objetivo.:**

Avaliar a soroprevalência de anticorpos contra a COVID-19 em indivíduos paucissintomáticos e assintomáticos, os fatores epidemiológicos associados e a cinética dos anticorpos da classe IgG em um período de 5 meses, visando aprimorar o conhecimento sobre a transmissibilidade da doença e a taxa de suscetíveis à infecção.

**Métodos.:**

Inquérito transversal de soroprevalência realizado na população geral (amostra não probabilística) de Santa Fé, na Argentina, entre julho e novembro de 2020. Um subgrupo de 20 indivíduos soropositivos foi acompanhado para analisar a persistência de anticorpos IgG. O kit de ensaio imunoenzimático (ELISA) COVID-AR IgG^®^ foi usado para a detecção de anticorpos IgG contra SARS-CoV-2.

**Resultados.:**

A amostra compreendeu 3 000 indivíduos, divididos entre assintomáticos e paucissintomáticos (n = 1.500 por grupo). Deste total, somente 8,83% (n = 265) apresentaram reatividade, com a detecção de anticorpos IgG contra SARS-CoV-2. Observou-se uma associação significativa entre a presença de anticorpos IgG contra SARS-CoV-2 e histórico de contato com caso confirmado. A taxa de transmissão intradomiciliar foi de aproximadamente 30%. No grupo paucissintomático, entre os soropositivos, o *odds ratio* (OR) para anosmia foi de 16,8 (IC 95% 9,5–29,8), e para febre, 2,7 (IC 95% 1,6–4,6) (p <0,001). Os indivíduos assintomáticos apresentaram níveis de IgG mais baixos que os paucissintomáticos, com uma tendência de declínio após 4 meses do início dos sintomas.

**Conclusões.:**

Observou-se uma soroprevalência baixa de anticorpos contra a COVID-19 na população estudada, o que indica um grande número de pessoas suscetíveis à infecção. Anosmia e febre foram preditores importantes independentes de soropositividade. Os assintomáticos apresentaram níveis mais baixos de anticorpos aos 5 meses de acompanhamento. Houve uma tendência de redução dos anticorpos IgG ao final deste período, independentemente da presença de sintomas.

To date, the reported surveys estimate that asymptomatic infection caused by SARS-CoV-2 ranges widely between 6% and 96% because of various conditions such as studies with low sample size or focused on specific subgroups as health personnel, follow-up of PCR-positive patients or their contacts (1). For this reason, these studies cannot provide accurate estimates of seroprevalence in the general population.

Characterizing the decline of specific antibody levels in asymptomatic/pauci-symptomatic individuals remains crucial to get a better knowledge of the disease transmissibility and the rate of susceptible persons that might be infected. For this reason, seroepidemiological studies play an important role. On the one hand, they show the proportion of the population exposed to the pathogen; on the other one, they allow to quantify the presence of antibodies against SARS-CoV-2 as a marker of total or partial humoral immunity, rendering possible to estimate the proportion of the sampled population that remains susceptible to the virus (2).

Describing the overall seroprevalence and the rate of actual cases allows a better assessment of the epidemic, hospitalizations, and deaths in a well-defined population helping to make some inference to a larger population (2, 3). Besides, the long-term kinetics of IgG antibodies against SARS-CoV-2 need to be further explored because current evidence is controversial. Wajnberg et al. (4) have recently described that antibodies against SARS-CoV-2 from mild to severe cases did not decline within 5 months since diagnosis. However, waning humoral immunity against SARS-CoV-2 has been reported in other studies within the 4 ensuing months (5).

Since the beginning of the pandemic, according to the World Health Organization (WHO) COVID Dashboard, until the 28^th^ of December, Argentina accumulated a total of 1.58 million cases. As per the national practice guidelines, the laboratory test to confirm the infection is performed if the patient has ≥2 symptoms compatible with COVID-19 disease or 1 symptom if the person has been in close contact with a confirmed case in the last 14 days. Also, the patient is tested if admitted to the hospital with a clinical presentation compatible with COVID-19 (6).

Within Argentina, the province of Santa Fe is one of the main locations presenting a major rate of confirmed cases of COVID-19. Since the beginning of the social isolation as a mandatory preventive health-policy in March 2020, until December 20^th^, the province accumulated a total of 130 000 laboratory-confirmed cases that were tested because of being symptomatic (7). Nevertheless, in the region and even at the national level, data from population-based seroprevalence studies have not yet been reported, so the real infection rate is probably underestimated (8, 9).

In this context, we evaluated the seroprevalence of COVID-19 infection in pauci-symptomatic and asymptomatic people, the epidemiological factors associated with this condition in the capital city of Santa Fe, along with the persistence of IgG antibodies against SARS-CoV-2 in a subset of samples.

## METHODS

### Study design and population

Seroprevalence was evaluated by a cross-sectional epidemiological study, based on the general population of the city of Santa Fe, with prospective inclusion of individuals through a non-probabilistic sampling (by convenience) from the beginning of July to late November 2020.

Volunteers over 18 years of age, of both sexes, residents from the different districts of the city were included. Individuals previously diagnosed with COVID-19 clinically and/or by laboratory tests (RT-PCR, antigen detection, or antibodies test) were excluded according to the criteria of suspected cases throughout the study period., we We also excluded people who had been in close contact with confirmed or suspected cases 20 days before taking the sample, if they developed symptoms attributable to COVID-19.

All people included in the study underwent an epidemiological interview based on the WHO instrument (2) for seroprevalence investigations, adapted to the study requirements, local setting, and outbreak characteristics. Sociodemographic and clinical data were collected: age, sex, telephone number, residence, number of people with whom they live, home characteristics, place of work, whether they have traveled abroad during January to March, trips outside the city and/or province, smoking and drinking habits, medical history and presence of symptoms related to COVID-19. To characterize the proportion of cases in which transmission occurred within household members, it was registered whether each participant was a cohabitant of a confirmed primary case of SARS-CoV-2. Possible intra-domiciliary transmission was considered in homes with ≥2 people living together and in which ≥1 of their members had presented positive diagnostic tests for SARS-CoV-2 infection.

Two subgroups of participants were distinguished (n = 1 500 each): i) individuals completely asymptomatic, and ii) individuals with pauci-symptomatic clinical features.

A subgroup of 20 seropositive individuals was followed-up to analyze the kinetics of IgG against SARS-CoV-2. The first sample was collected after 50 to 60 days since their cohabitant was diagnosed with COVID-19 disease by RT-PCR test, whereas 3 additional samples were taken approximately every 30 days.

The study was carried out following the ethical principles for research with human beings established in the Nüremberg Code, the Belmont Report, and the Declaration of Helsinki. The protocol was registered and approved by the Provincial Bioethics Committee of the Province of Santa Fe (RP N°953) and by the Bioethics Committee of the FCM (UNL). All subjects received complete information about the study and signed an informed consent. Patients were assigned codes in the databases in order to safeguard the confidentiality of their personal data.

### Sample processing and detection of specific anti-SARS-CoV-2 IgG by ELISA

After the epidemiological interview, a blood sample was extracted by venipuncture from each patient for the serological test (5 ml), which was preserved with sodium heparin, labeled with a unique code. The collected samples were centrifuged at 2 500-3 000 rpm for 10 minutes for extracting the serum and stored in 1.5 ml microtubes in a refrigerator (2-8 °C) until its employment for antibody detection assays within the 7 following days.

For the detection of IgG, we employed the COVID-AR IgG^®^ Kit (developed by Leloir Institute, Lemos Laboratory, and the National University of San Martín) authorized by the Argentinian National Drug Administration, Food and Medical Technology (Medical Product 1545-4) (10). It consists of a heterogeneous, non-competitive, indirect-type immunoenzymatic assay for the quantification of IgG antibodies against SARS-CoV-2 antigens with a specificity of 100% and a sensitivity greater than 96.7% in samples from 21 days after onset of symptoms (11,12). The kit provides 96-microwell polystyrene plates coated with recombinant spike protein (S1) and RBD (receptor binding domain), the specific fragment containing the human ACE2 receptor binding site. Serum samples were prepared at 1/51 dilution in the sample buffer, placing 200 μl/well. After incubation at 37 ºC for 1 hour, 6 manual washing cycles were carried out. Then 100 μl/well of a solution composed of anti-human IgG antibody conjugated to peroxidase at a dilution of 1/10 was incubated for 30 minutes. After a new cycle of washing, 100 μl/well of chromogen was applied for 10 minutes, interrupting the reaction with 100 μl/well of 2N sulfuric acid solution. The absorbance was quantified in a microplate reader at 450 nm wavelength (EMP M201 Microplate Reader).

Each sample was evaluated in duplicate, identifying as positive those samples whose mean optical density (OD) exceeded the cut-off point (0.200 + mean OD of negative controls). As negative and positive controls, we used those provided by the manufacturer. The variation between the assays was covered by taking at least 6 negative controls on each plate. In addition, the average value obtained was compared to that reported for negative controls in the manufacturer's instructions (OD ≤0.264). This was fulfilled in all cases, reflecting scarce inter-assay variation that allows comparison (average cut-off: 0.216 ±0.008).

### Statistical data processing and analysis

Data were analyzed with IBM SPSS Statistics v24.0 (Armonk, NY: IBM Corp.). The distribution of quantitative variables was evaluated using Kolmogorov-Smirnov test. The normally distributed results were presented as mean ± standard deviation (SD) and were analyzed by two-way T-tests (for unpaired data) or by ANOVA followed by an appropriate *post hoc* test. Those who did not meet the normal condition were presented as median with interquartile range (IR) and analyzed with non-parametric tests (Mann-Whitney or Friedman tests). Differences between proportions were evaluated by using *x*^2^ test. A p-value <0.05 was considered significant for two-tailed tests. A multiple binary logistic regression model step forward conditional with the variables associated with seropositive results was performed to assess which ones were predictive of the presence of IgG anti-SARS-CoV-2.

## RESULTS

### General sample

A total of 3 000 participants were included, with 60.1% (n= 1 803) being women. The mean age was 39.4 ±12.5 years, showing no sex-related differences. Most of the sample was composed by people between 18 and 45 years (70.6%, n=2 118), a 25.3% (n=749) of the individuals were aged between 46 and 65 year-old, and the remaining (4.1%, n=133) by older adults.

Table 1 summarizes medical history and/or comorbidities from subjects. Of the total number of participants, 74.7% (n=2243) did not refer to any relevant pathological condition on their medical history. Arterial hypertension (21.7%), hypothyroidism (22.1%), asthma (13.5%), and diabetes (7.0%) were the most prevalent conditions.

When assessing whether the individuals had been in contact with a confirmed case of SARS-CoV-2 infection, 64.8% denied any contact, whereas the remaining ones referred so, 11.1% at the workplace, 10.3% at home (cohabiting), 6.7% in social meetings, 5.4% in family meetings, and 1.2% of them in sports center, commerce and gastronomic services.

### Seropositive individuals

From the total sample, only 8.83% (n=265) presented reactivity for IgG antibodies against SARS-CoV-2. The age of the seropositive people was 39.7 ±13.8, with no between-sex differences. Most of the seropositive individuals were aged between 18 and 45 years (70.6%; n=187), 26.2% (n= 69) between 46 and 65 years, whereas 3.2% (n=9) were older than 65 years. Taking into account the age distribution of the whole sample, the positivity rate within each group was similar, 8.2%, 9.9%, and 10.9%, respectively).

From the total of seropositive patients, 40.1% individuals denied close contact with confirmed cases of COVID-19 disease. On the other hand, 25.7% reported having had contact with a confirmed case at home (cohabiting), 18.1% in the context of a social meeting, 8.3% at the workplace, and 6.3% in family meetings. Among patients that referred contact with a confirmed case at home, only 21.7% resulted seropositive.

**TABLE 1.** Distribution of most frequent comorbidities reported by 3 000 individuals

**Comorbidities**	**Asymptomatic (n=1 500)**		**Pauci-symptomatic (n=1 500)**		***p***	**TOTAL (n)**
	**%**	**N**	**%**	**N**		
Without comorbidities	76.9	1153	72.7	1 090	0.008^*^	2 243
Hypothyroidism	4.7	70	6.5	97	0.03^*^	167
Hypertension	6.3	95	4.7	70	0.05^*^	165
Asthma	2.5	37	4.3	65	0.006^*^	102
Diabetes	1.6	24	2	29	NS	53
History of neoplastic disease	1.1	16	2.1	31	0.02^*^	47
Hypertension, hypothyroidism	1.2	17	0.9	14	NS	31
Hypertension, diabetes	0.8	11	1	16	NS	27
Other disease	0.9	12	0.4	6	NS	18

* *x*^2^ test.

NS, non significant.

The asymptomatic group (n=1 500) was 40.12 ± 12.3 years old and 56.5% were female. Only 4.8% (n=72) of them had anti-SARS-CoV-2 IgG antibodies. People from the pauci-symptomatic group (n=1 500) had a rather similar age (38.69 ± 12.7 years) and sex distribution (64.4% women). In this group, the seroprevalence was significantly higher, 12.9% (n=193; p <0.001). Both groups showed no differences as to age or sex distribution when comparing positive and negative cases. Among the seropositive cases, the proportion of asymptomatic individuals was comparable among the above defined three age groups (27.8%, 36.4%, and 33.3%, respectively).

**TABLE 2.** Distribution of epidemiological contact with a confirmed case of COVID-19 reported by sampled individuals on asymptomatic, symptomatic groups and among those who resulted seropositive

**Contact**	**Asymptomatic**		**Symptomatic**		***p***	**Seropositive**	
	**%**	**N**	**%**	**N**		**%**	**N**
Denied close contact	59.7	896	69.8	1 047	<0.0001	40.1	106
Workplace	12.8	192	9.8	146	0.009	8.7	23
Home (cohabiting)	11.1	167	9.5	143	NS	25.7	69
Social meetings (friends)	8.9	133	4.9	74	<0.0001	17.7	47
Familiar meetings	5.9	88	4.9	74	NS	6.10	16
Sport centers	0.7	10	0.8	11	NS	0.75	2
Bar, restaurant	0.5	8	0.2	3	NS	-	-
Shops	0.4	6	0.1	2	NS	0.75	2
Total		**1 500**		**1 500**			**265**

NS, non-significant.

The distribution of comorbidities within each group (symptomatic/pauci-symptomatic) did not deviate from the one recorded in the whole sample (Table 1), with a higher proportion of hypertension (p= 0.05), hypothyroidism (p= 0.03), and asthma (p= 0.006) in the pauci-symptomatic group.

We observed a significant association between the presence of IgG anti-SARS-CoV-2 and a previous contact history with a confirmed case of SARS-CoV-2 infection compared to seropositive patients who denied any close contact (59.9% vs 40.1%; p= 0.001). The situation in which the contact with the confirmed case took place in each group is shown in Table 2. In the asymptomatic group, a higher proportion of subjects reported a contact history in the workplace or in social meetings, while in this group fewer of them denied known contact.

Besides this, cohabiting with a confirmed case and social meetings were significantly associated in both groups with presenting seropositivity (p <0.001 for both).

Of 137 family groups analyzed, 63 of them met criteria for possible secondary household transmission of SARS-CoV-2, which was found only in 14/63 (22.2%) of groups of cohabitants studied in our sample being in most cases pauci-symptomatic (9/14).

In the asymptomatic group, a logistic regression model was employed to assess the presence of anti-SARS-CoV-2 IgG including variables such as contact with a confirmed case and the individual circulation characteristics (attending to work, shopping, assisting a relative or acquaintance, if any of the cohabitants performed any of these activities, and the mobility used). Nevertheless, none of them contributed significantly to the prediction of seropositivity among asymptomatic subjects.

Regarding the clinical presentation in pauci-symptomatic patients, Table 3 shows the 10 most prevalent referred symptoms. The most common symptoms in pauci-symptomatic seropositive cases (n=193) were anosmia in 42.4% (n=82), headache in 41.4% (n=80), fever (>37.5 °C) in 36.4% (n=70) and muscle pain in 33.3% (n=64). When analyzing the different symptoms to ascertain their relationship with seropositivity (by odds ratio), anosmia presented an OR of 16.8 (95% CI 9.5-29.8) whereas fever (>37.5 °C) an OR of 2.7 (95% CI 1.6-4.6, p < 0.001 in both cases). Consistently, the model for predicting seropositivity including both symptoms shows an area under the curve of 78% (95% CI 74.9-80.9; p <0.0001).

When incorporating variables like contact with a confirmed case of SARS-CoV-2 infection and individual circulation into the analysis, the model did not vary significantly, either for anosmia (OR 16.9, 95% CI 9.4-30.5) or fever (OR 2.6, 95% CI 1.5-4.4). Regarding the variable of contact with a confirmed case, the option at home (cohabiting) yielded an OR of 2.7 (95% CI 1.4-5.3) and social meeting an OR of 2.6 (95% CI 1.3-5.2). Upon including these variables, the area under the model curve was 80% (95% CI 77.5-83.3) without significant variation compared to the previous model.

**TABLE 3.** Clinical presentation of the pauci-symptomatic individuals and seropositive cases among them (only the 10 most frequent symptoms, or their association are enlisted)

**Symptoms in pauci-symptomatic individuals**	**%**	**n=1 500** N
Headache	15.8	237
Cough	7.8	117
Cold	6.7	100
Headache, cold	4.2	62
Sore throat	4.2	62
Headache, muscle pain	3.4	51
Fever (≥37.5 °C)	3.0	45
Fever (≥37.5 °C), cough	2.4	35
Anosmia	2.0	29
Diarrhea	1.8	27
Headache, diarrhea	1.8	27

**Symptoms in seropositive pauci-symptomatic individuals**	**%**	**n=193** N
Anosmia	11.0	21
Headache, cough, anosmia, ageusia, muscle pain	10.0	19
Headache	7.0	13
Fever (≥37.5 °C)	5.0	10
Fever (≥37.5 °C), anosmia	5.0	9
Fever (≥37.5 °C), headache, anosmia, muscle pain, asthenia	5.0	9
Headache, muscle pain	3.0	5
Diarrhea	2.0	4
Anosmia, ageusia	2.0	4
Headache, anosmia	2.0	3

**FIGURE 1.**
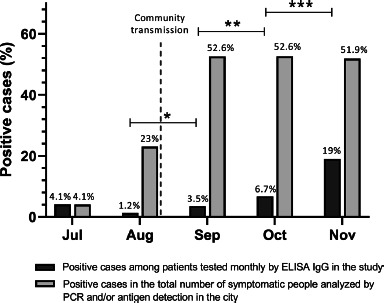
Monthly rate of seropositive cases determined by ELISA in the study sample and rate of confirmed cases in the general population by PCR or antigen tests

Note: Absolute values of seropositive test in the total ELISA assays performed per month: July = 4/96; August = 6/469; September = 17/475; October = 74/1097 and November = 164/863. Absolute positive cases among the total of PCR tests performed per month: July = 19/461; August = 431/1870; September = 2493/5590; October = 4689/8902 and November = 5619/10825. The dashed line indicates the date wherein community transmission was declared in the city (8/28/20). T test for proportions: * p = 0.019; **p = 0.012; *** p ≤ 0.0001.

Concerning the number of tests carried out per month (Figure 1), we observed a progressive increase in the seropositivity rate from September 2020 (17/475), being significantly higher in November 2020 (164/863). This trend is in line with an increase of the laboratory-confirmed cases from the general population due to the local circulation of the virus declared in August 2020. According to the official reports from Santa Fe Province Ministry of Health, in July 2020 the rate of positivity by rapid antigen detection and/or RT-PCR methods was 4.1% (19/461), further increasing significantly to 23.1% (431/1870) in August 2020 (p <0.0001). However, from September to November the positivity rate was slightly over 50%.

From July until the end of November 2020 the total symptomatic cases that met the criteria to be tested were 27 648, with 13 251 (47.9%) confirmed cases.

### Persistence of IgG against SARS-CoV-2

**FIGURE 2.**
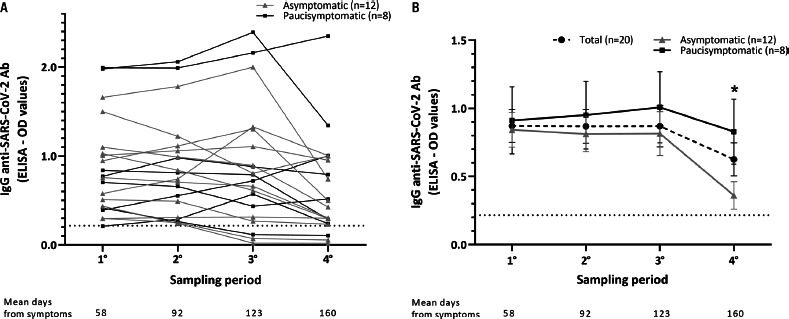
Levels of anti-SARS-CoV-2 IgG antibodies of 20 subjects throughout the 5-month follow-up. A) Individual levels of IgG. For each patient, the mean value obtained in the duplicates from the sample corresponding to the different follow-up periods is represented. B) Average levels of anti-SARS-CoV-2 IgG of followed-up subjects according to their clinical presentation (pauci-symptomatic or asymptomatic).

A subgroup of 20 seropositive patients (Figure 2A) were followed-up to analyze whether antibodies persisted 3 months after the infection.

The mean interval between the first antibody measurement and the second one was 58 days (range: 31-70). Consequently, the second sample was taken at a mean of 92 days after symptom onset (range: 78-99 days), the third one at 123 days (range: 110-135 days), and the last one at 160 days (range: 148-170). In most cases, antibody levels remained positive over the study time. Only in 3 cases, serological negativization was observed in the third sample. It should be noted that 2 of these patients were asymptomatic while one was pauci-symptomatic; all of them presented low antibody values at the beginning of their follow-up (mean OD: 0.383 ±0.074). There were also 3 patients in the pauci-symptomatic group who increased their antibody levels during follow-up.

Ab, antibodies; OD, Optical density.

Note: The box plots represent the mean ± SD of the OD value obtained among studied patients together and within each group. The dotted line indicates the average value of the cut-off point obtained between successive assessments of the IgG antibodies by ELISA method (OD 0.216). * Friedman test: p = 0.0046; Mann-Whitney U test: p = 0.045.

When performing an analysis by sex, no significant differences were observed in the IgG values during the follow-up at any of the studied times. As can be seen in Figure 2B, although average antibody levels tended to be higher in the symptomatic patients if compared to asymptomatic ones, the difference was not statistically significant during the first months. Nevertheless, after a 4-month follow-up, the mean values began to decline significantly, being this decrease specially marked in the asymptomatic group.

## DISCUSSION

Due to the fact that official case counts are based on tests carried out in symptomatic individuals and the absence of seroprevalence data from population-based studies in Argentina, the actual number of people who have been infected with SARS-CoV-2 is probably underestimated (8, 9). In this context, this is the first reported research in Argentina including general population, providing a regional estimation of the presence of anti-SARS-CoV-2 IgG antibodies in asymptomatic and pauci-symptomatic people over a full 5-month period of the pandemic. Different techniques can be employed to assess the rate of asymptomatic COVID-19 infection. We used indirect ELISA (COVID-AR^®^) due to its availability and high sensitivity and specificity (12). Rapid lateral immunochromatography tests may be an alternative, providing easy-to-read qualitative results, but with the disadvantage of providing false positive and false negative results. On the other hand, chemiluminescence immunoassays could be used, but require more sophisticated equipment.

In this subset, we observed a low seroprevalence rate (8.83%). In Argentina, only two studies assessed seroprevalence outside the setting of health care workers, both carried out in slums of the city (9) and the province of Buenos Aires (13), reporting seropositivity of 14% and 50%, respectively, over a sampling period of less than a month. However, as mentioned above, no other studies were carried out, limiting the generalization of these results to the rest of the country given the demographic differences. Although our sample was non-probabilistic due to feasibility and resource reasons, we were able to include a wide age range and individuals from all city districts with a similar sex distribution.

As stated by Wells et al. (14), defining the seroprevalence of COVID-19 is crucial in predicting the course of the disease and the likelihood of sustained transmission. It also helps to estimate the development of herd immunity and the influence of vaccination. Our findings indicate a low seroprevalence rate among asymptomatic and pauci-symptomatic cases, which is consistent with the average prevalence of COVID-19 reported by Yanes-Lane et al. (15). Besides, we observed that for every ~3 seropositive pauci-symptomatic people, there was one seropositive case fully asymptomatic (193 pauci-symptomatic seropositives/72 asymptomatic seropositives=2.68); 27.7% of infected individuals experienced no symptoms. Similarly, a narrative review by Oran et al (1) described that approximately 40% of individuals infected with SARS-CoV-2 are asymptomatic.

However, an increased seroprevalence was seen near the end of the sampling period, which may be a consequence of the local circulation of the virus and the relaxation of social distancing measures (16). The application of the health policy to change from Obligatory Preventive Social Isolation to Obligatory Preventive Social Distancing was in mid-June 2020. However, recreational, commercial activities and social gatherings began to be gradually enabled in late September 2020; this coincides with the gradual onset of increase in the seropositivity rate.

It is worth mentioning that in the case of asymptomatic people, the logistic regression model did not show a predictive association from epidemiological contact or circulation with seropositivity. These results may be due to the fact that almost half of the asymptomatic patients do not identify contact with an infected person; in addition, 78.3% of the subjects who referred contact with a confirmed case at home were seronegative, which is accompanied by a 30% within-household positivity rate. It should be clarified that this data corresponds only to adult cohabitants since no children were evaluated; the result is consistent with previous studies reporting a rate of secondary attack of the infection between 16% and 53% (17-19).

In the pauci-symptomatic group, the epidemiological data about contact with a confirmed case yielded a relevant OR for seropositive in the cases of cohabiting (OR 2.7) and social meeting (OR 2.6). However, when analyzing the symptoms, the 16.8 OR of anosmia was considerably substantial. Anosmia plus fever resulted in an area under the curve for predicting seropositivity of 78%, that remained so when adding epidemiological data. This result is in line with the study from Pierron et al. (20) stating that smell changes should be used as an indicator of COVID-19.

When following up a subgroup of symptomatic or pauci-symptomatic patients over several months, it was clear that seropositivity persisted, but IgG levels tend to decrease in most cases. These results are consistent with reports from other locations, indicating that humoral immunity does not appear to be long-lasting in people with mild or asymptomatic disease (5, 21, 22). In the case of the 2 pauci-symptomatic patients who increased their IgG values starting from relatively low levels, it would be expected that in subsequent months they would tend to decrease or even become negative, perhaps faster than what could be expected from the third case that had a high value in the first sample and it continues to rise until the fourth evaluation.

It should be noted that antibody levels from asymptomatic patients were, on average, significantly lower than those yielded by patients who had mild symptoms at 5 months of follow-up. In this sense, Ojeda et al. quantified anti-SARS-CoV-2 IgG antibodies in 40 asymptomatic and 40 symptomatic patients from Argentina. Using the same ELISA kit, they observed that asymptomatic cases exhibit mean levels that were below those observed in their series of symptomatic patients (12). Recently, García-Beltrán et al reported a similar trend in 98 patients evaluated over 2 months; subjects with milder symptoms had lower titers and less neutralizing activity of antibodies, compared to those with more severe COVID-19 (23). Our study extends such results, by also providing follow-up information for a longer period.

In conclusion, our study provided evidence that among pauci-symptomatic patients, anosmia and fever were independent significant predictors for seropositivity. Also, during the follow-up of asymptomatic patients, their antibody levels were lower than those achieved by pauci-symptomatic patients, tending to decline significantly after 4 months. Since seropositivity rates can be used as a marker of humoral immunity, serving to estimate the proportion of people that remain susceptible to the virus, the low seroprevalence seen in this sample may imply a high population susceptibility to SARS-CoV-2 in the study region.

## Disclaimer.

Authors hold sole responsibility for the views expressed in the manuscript, which may not necessarily reflect the opinion or policy of the RPSP/PAJPH and/or PAHO.
